# Epithelioid Sarcoma of the Spine: A Review of Literature and Case Report

**DOI:** 10.3390/jcm12175632

**Published:** 2023-08-29

**Authors:** Yi Liang Tan, Wilson Ong, Jiong Hao Tan, Naresh Kumar, James Thomas Patrick Decourcy Hallinan

**Affiliations:** 1 Department of Diagnostic Imaging, National University Hospital, 5 Lower Kent Ridge Rd, Singapore 119074, Singapore; prooyf@hotmail.com (W.O.); james_hallinan@nuhs.edu.sg (J.T.P.D.H.); 2 University Spine Centre, Department of Orthopaedic Surgery, National University Health System, 1E, Lower Kent Ridge Road, Singapore 119228, Singapore; jonathan_jh_tan@nuhs.edu.sg (J.H.T.); dosksn@nus.edu.sg (N.K.); 3 Department of Diagnostic Radiology, Yong Loo Lin School of Medicine, National University of Singapore, 10 Medical Drive, Singapore 117597, Singapore

**Keywords:** ES, spine, intradural, clinical outcome

## Abstract

Epithelioid sarcoma is a rare malignant mesenchymal tumor that represents less than 1% of soft-tissue sarcomas. Despite its slow growth, the overall prognosis is poor with a high rate of local recurrence, lymph-node spread, and hematogenous metastasis. Primary epithelioid sarcoma arising from the spine is extremely rare, with limited data in the literature. We review the existing literature regarding spinal epithelioid sarcoma and report a case of epithelioid sarcoma arising from the spinal cord. A 54 year old male presented with a 1-month history of progressive left upper-limb weakness and numbness. Magnetic resonance imaging (MRI) of the spine showed an enhancing intramedullary mass at the level of T1 also involving the left T1 nerve root. Systemic radiological examination revealed no other lesion at presentation. Surgical excision of the mass was performed, and histology was consistent with epithelioid sarcoma of the spine. Despite adjuvant radiotherapy, there was aggressive local recurrence and development of intracranial metastatic spread. The patient died of the disease within 5 months from presentation. To the best of our knowledge, spinal epithelioid sarcoma arising from the spinal cord has not yet been reported. We review the challenges in diagnosis, surgical treatment, and oncologic outcome of this case.

## 1. Introduction

Epithelioid sarcoma (ES) is a rare malignant mesenchymal neoplasm that represents <1% of all sarcomas [1]. First described by Enzinger in 1970, it was characterized as a slow-growing painless swelling typically occurring in the distal extremities [2]. It is challenging to diagnose ES accurately, as it is known to mimic a chronic inflammatory process which is often misdiagnosed as a benign condition clinically [2,3,4]. Despite the often-indolent clinical presentation, the disease has a poor prognosis, with high propensity for local recurrence, as well as lymphatic and hematogenous metastatic spread [5]. Primary ES arising from the spine is extremely rare, with limited data in the literature [6,7]. This report aims to review the published literature regarding ES and in particular spinal ES, with attention to its clinical presentation and behavior, challenges in initial diagnosis, treatment, and outcomes. In addition, we report a case of intraspinal ES arising from the spinal cord and its outcome.

### 1.1. Epithelioid Sarcoma: Natural History and Presentation

ES is a rare malignant soft-tissue neoplasm which is currently divided into two distinct clinical and morphological subtypes according to the 2020 WHO classification of tumors of the soft tissue and bone: the conventional form or classic type of ES (also known as the “distal type”) and the proximal type [8]. The terms distal and proximal initially indicated the anatomical location of the tumor; however, it now has histological significance regardless of the site of the tumor [9]. The two subtypes have differing clinical characteristics.

The classic type of ES typically presents as a slow-growing painless soft-tissue swelling in the distal extremities of young adults, often associated with ulceration of the overlying skin. The classic type of ES is rare in children and the elderly, and it most commonly occurs in young males (up to a 2:1 male to female ratio), with an age range from 10 to 45 years old [9,10]. It has a tendency for local recurrence and propensity for regional lymph-node metastasis and distant metastatic spread to the lungs, brain, and bone [11].

The other less common subtype is termed the proximal subtype and is clinically characterized by its more axial distribution, involving the deep soft-tissue structures of the proximal limbs, limb girdles, and midline of the trunk. The typical presentation is an infiltrative mass in the deep soft tissue which can attain a large size (up to 20 cm in diameter) [5,11]. The median age of presentation is slightly older than the classical subtype (40 years vs. 30 years of age) [12]. The proximal subtype also tends to have a have a more aggressive clinical behavior from the outset, with higher rates of local recurrence and earlier metastatic spread [9,11,13].

### 1.2. Epithelioid Sarcoma: Histopathology

On microscopic examination, the classic type of ES is characterized by irregular nodular proliferation of epithelioid cells with relatively abundant eosinophilic cytoplasm and minimal pleomorphic nuclei [11]. They are composed of relatively uniform polygonal cells, often with loss of cellular cohesion, and they merge imperceptibly with spindle cells at the periphery of the tumor [5]. The nodules also typically demonstrate central necrosis, which can simulate a granulomatous process [14]. On the other hand, the proximal type of ES demonstrates a multinodular pattern of larger polygonal cells with pleomorphic vesicular nuclei and prominent nucleoli [15,16]. Focal or predominant rhabdoid features are often present in this subtype [9,15]. Frequently, there is also central necrosis, although this may be with or without the geographic pattern of necrosis, which is typical of the classic type of ES. Rarely, a tumor may have features of both the classic and the proximal variants [5].

No single specific tumor marker for diagnosis of ES has been established. Virtually all cases of ES demonstrate cytokeratin (CK) and epithelial membrane antigen (EMA) positivity. Most cases co-express vimentin and CK, but a few are vimentin-negative [5]. The immunophenotype of diffuse CK and EMA expression is similar to metastatic carcinoma, with which ES is most often confused with on histology. In contrast to carcinoma, CD34 staining is positive in more than half of ES, and the combination of CD34 and EMA expression can help to differentiate them [5,14].

ES also demonstrates unique cytogenetic findings, with a loss of INI-1/SMARCB1 expression. INI1 (also known as hSNF5 and SMARCB1) is a remodeling complex located on the long arm of chromosome 22 that is ubiquitously expressed in the nuclei of all normal cells. Loss of SMARCB1/INI1 expression is seen in >90% of both conventional and proximal types of ES [14,17]. However, this is not specific for ES, as loss of SMARCB1/INI1 is also seen in other malignancies including renal medullary carcinoma, malignant rhabdoid tumor, and epithelioid malignant peripheral nerve sheath tumor (MPNST) [17].

### 1.3. Epithelioid Sarcoma: Diagnosis

As ES often presents as a slow-growing indolent tumor and is rarely encountered in clinical practice, it can be initially misdiagnosed as a benign condition [5]. It resembles both non-neoplastic lesions and benign neoplasms of the skin and soft tissue. As was noted in the original paper by Enzinger, it was often clinically thought to be an inflammatory process, and alternative diagnoses were considered after a lack of expected response to treatment. The role of imaging in the diagnosis of ES is limited, and definitive diagnosis is, thus, based on tissue histopathology; however, this can be challenging due to ES having many histopathologic mimics. Even with biopsy and microscopic examination, the malignant potential of the tumor is not always apparent, as it may have a relatively bland cytology and pseudogranulomatous appearance [11]. On microscopy, benign mimics include granuloma annulare, necrobiosis lipoidica, a chronic granulomatous inflammatory process (for example, rheumatoid nodules), nodular fasciitis, and fibromatosis [5,11]. However, the presence of mitotic activity should raise the index of suspicion for ES. In addition, the presence of diffuse epithelial marker positivity should also point toward the right diagnosis [11,18]. As ES can sometimes comprise spindle-shaped cells, malignant mimics include those of malignant spindle-cell neoplasms such as synovial sarcomas, fibrosarcomas, angiosarcomas, malignant fibrous histiocytomas, and malignant extrarenal rhabdoid tumors [11,14,18]. The proximal type of ES should also be differentiated from other neoplasms with epithelioid and/or rhabdoid cells, for example, extrarenal malignant rhabdoid tumor, melanoma, rhabdomyosarcoma, malignant peripheral nerve sheath tumor, undifferentiated carcinoma, and other sarcomas including synovial sarcoma and extraskeletal myxoid chondrosarcoma [2,3,5]. Appropriate immunohistochemical staining showing positivity for vimentin, cytokeratins, EMA, and CD34, and negativity for the S100 protein, HMB-45, FLI-1, and desmin, as well as SMARCB1/INI1 gene deletion, is helpful to arrive at an accurate diagnosis and exclude most of the aforementioned tumors [15].

### 1.4. Epithelioid Sarcoma: Evaluation, Treatment, and Prognosis

Due to the rarity of ES, a consensus regarding the optimal evaluation and treatment strategy for ES is not established [13]. After definitive histopathological diagnosis, staging and evaluation should be performed with particular focus on assessing regional lymph-node and pulmonary involvement. Lymph-node metastases may be an early symptom of widely disseminated disease rather than a locoregional process [19], and further evaluation of regional lymph nodes with ultrasound scanning in combination with guided fine-needle aspiration may be helpful in staging [20].

ES is an aggressive neoplasm, often recurring persistently and eventually metastasizing [13,21]. Recurrence is often noted to occur, with a recurrence rate varying from 34% and 77% depending on the adequacy of initial excision [10,11,20]. Metastasis occurs in 40% of patients, predominantly involving the lungs, regional nodes, scalp, bone, and brain [20,21].

Currently, when the mass is localized, wide surgical resection is regarded as the only curative modality for treatment of ES [15,20]. Initial diagnosis of the tumor as benign may lead to positive resection margins and delayed diagnosis, affecting the prognosis [22,23]. Poor prognostic factors for ES in the literature include a proximal or axial tumor location, increased size and depth, hemorrhage, mitotic figures, necrosis, presence of vascular invasion [10], and the presence of distant metastases at the time of diagnosis [24].

There is a reported potential benefit of adjuvant radiotherapy in reducing recurrence after surgical resection, and radiotherapy is routinely used as an adjuvant to surgery [15,24,25]. In a retrospective multicenter Korean study, adjuvant radiotherapy was noted to significantly prolong recurrence-free survival [13].

Perioperative therapy for ES is often used in a wide range of cases, but mainly when the tumor is large or high-grade, or in cases with metastases [14]. Regimes in the literature include doxorubicin as monotherapy or VAIA (vincristine, doxorubicin, ifosfamide, and actinomycin-D) and CyVADIC (cyclofosfamide, vincristine, doxorubicin, and dacarbazine). A combination of doxorubicin and ifosfamide was most commonly used in studies from the Royal Marsden Hospital, Japan, and French Sarcoma Group [15]. In contrast to post-operative radiotherapy, adjuvant chemotherapy does not appear to confer a clear benefit, and its role in the treatment of ES is less established [13,24].

### 1.5. Epithelioid Sarcoma of the Spine

Primary spinal ES is very rare, with only a few published case reports and case series. One systematic review of primary spinal ES was published by Hu W. et al. and involved 15 cases of spinal ES. In that review, five cases were in the cervical spine, nine cases were in the thoracic spine, and another nine cases were along the lumbar spine. The location of the spinal tumor affected survival time, with those in the lumbar spine having the shortest survival time. The survival time for lesions in the lumbar vertebra was 10.4 ± 6.6 months, while survival times for lesions in the thoracic and cervical vertebra were 27.9 ± 14.6 months and 17.0 ± 13.8 months, respectively. There was a male predominance (2.29:1 male-to-female ratio) and the mean patient age was 33.7 years, consistent with ES elsewhere [26]. In another review of seven cases by R. Babu et al., the average patient age was 20.7 years, and 71.4% of the patients (5/7) were male [27]. According to the WHO classification of ES, only one case in the review by Hu W. et al. was classic type, while all other cases were the proximal type.

Imaging of spinal ES was noted to be variable on MRI imaging, with tumors appearing hypo- or isointense on T1-weighted imaging, showing variable intensity on T2-weighted imaging, and demonstrating avid contrast enhancement [27]. The average diameter of the mass was 6.8 ± 3.1 cm, and the size distribution ranged from 3.9 to 15.0 cm [26]. The mass often extends into the spinal canal, resulting in spinal-cord injury [26,27]. Metastasis at presentation was also common and found in up to 83.3% of the patients, with the most common being lung metastasis [26]. Treatment of spinal ES is more challenging than in other parts of the body due to surrounding neural structures. This is even more so in the cervical spine where involvement of the vertebral arteries further increases the risks and complexity of the surgery. The goal of surgery is often to achieve gross total resection of all the tumor and involved bony elements if feasible, without significant neurological deficits [6]. Resection of the nerve roots may also be required as nerve sheaths can serve as pathways for tumor spread [6,27]. Several authors suggest spondylectomy and en bloc resection of the tumor as the best option to eradicate disease and prevent recurrence [28]. However, as ES of the spine often has spinal canal invasion and involvement of neural structures, intralesional resection is often the only option [6,28,29]. In a case of recurrent ES of the thoracic spine by Chanplakorn et al., repeat radical surgery with en bloc resection of the spinal mass with the involved spinal implants and sacrifice of the spinal cord allowed for a local recurrent-free period of 26 months [28]. This suggests that initial radical resection of ES in the spine remains the best treatment option.

Post-operative radiotherapy and chemotherapy were also noted to provide improved survival times in spinal ES in the aforementioned systematic review [26]. This is in contradistinction to ES in the rest of the body, where adjuvant chemotherapy has no influence on relapse-free survival [13]. The most common chemotherapy regime used in spinal ES is that of ifosfamide + doxorubicin [26].

The United States Food and Drug Administration (USFDA) recently granted accelerated approval to tazemetostat for the treatment of adults and pediatric patients aged 16 years and older with metastatic or locally advanced ES not eligible for complete resection in January 2020. As mentioned above, loss of INI1 expression is seen in the majority of ES. When INI1 loses its regulatory function, there is increased expression and recruitment of EZH2, a potent histone methyltransferase, which is the enzymatic component of a protein complex, ultimately leading to increased methylation of histone H3K27. This leads to upregulation of several oncogenic pathways, including Sonic Hedgehog, Wnt/β-catenin, and MYC [30]. Tazemetostat is an inhibitor of EZH2 and works by preventing the methylation of histone H3K27. It has a good safety profile and has shown substantial clinical benefit in patients with advanced ES. The overall response rate of tazemetostat was 15% in a first=line setting and 8% in those with prior systemic therapies in a phase 2 clinical trial for tazemetostat in INI1-negative tumors (NCT02601950) [15,30]. As spinal ES is prone to difficult surgical resection, tazemetostat represents a new therapeutic option. There is an ongoing phase 1b/3 trial investigating tazemetostat as a front-line therapy for patients with advanced ES [30].

With regard to immunotherapy using immune checkpoint inhibitors (ICIs), there have been some case reports demonstrating a response ranging from partial remission to complete remission. These case reports used anti-PD1 ICIs such as pembrolizumab or nivolumab, or anti-CTLA-4 ICIs such as ipilimumab [31,32]. However, another retrospective analysis from a tertiary care center in India found that immunotherapy in two patients did not have any objective response [33].

## 2. Case Presentation

A 54 year old Chinese male presented to the emergency department with a 1-month history of progressive weakness and numbness of the left upper limb. Apart from diabetes mellitus, he had no significant past medical history. At the time of admission, his neurological examination revealed left upper-limb weakness, with a motor grade of 3 out of 5 for his left upper extremity. There was also hypoesthesia of the left upper limb involving the left C8 and T1 distribution. No increased deep tendon reflex was elicited.

**Figure 1:**
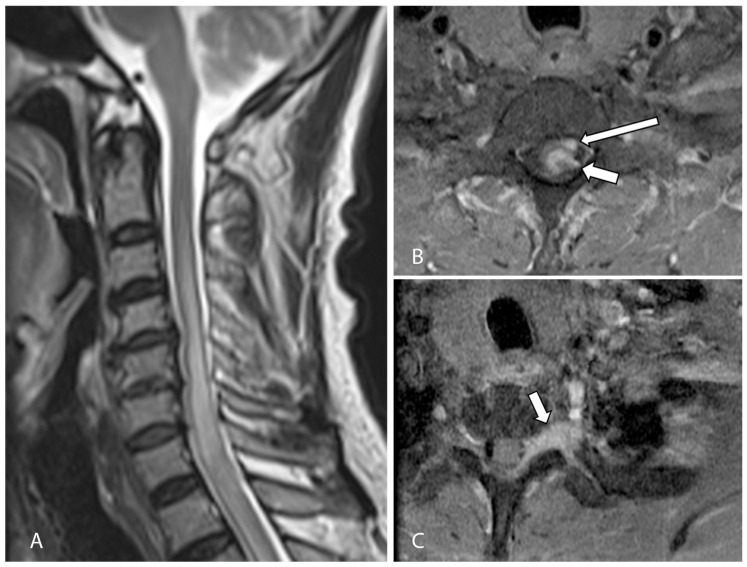
(**A**) Initial sagittal T2-weighted MRI of the cervical spine showed long segment cord signal change/edema and cord expansion. (**B**) Axial T1-weighted, fat-suppressed, contrast-enhanced MRI of the cervical spine shows an enhancing intramedullary lesion at the level of T1 (short arrow). There is also a nodular enhancing focus which appeared extramedullary in location (long arrow). (**C**) Axial T1-weighted, fat-suppressed, contrast-enhanced MRI of the brachial plexus at a level slightly inferior to (**B**) shows abnormal thickening and enhancement along the left T1 nerve root (arrow), suspicious for perineural disease spread.

Magnetic resonance imaging (MRI) of his cervicothoracic spine and left brachial plexus demonstrated a 1.3 × 0.7 × 0.8 cm intraspinal mass centered at the level of T1 on the left. This lesion was heterogeneous and infiltrative, with poorly defined margins. It had an iso- to hypointense T2-weighted signal and demonstrated heterogeneous contrast enhancement. It appeared predominantly intramedullary in location with associated expansion and edema of the spinal cord. However, there was also thickening and enhancement along the left T1 nerve root with expansion of the left neural foramina, as well as a nodular enhancing focus which appeared extramedullary in location (Figure 1). On the basis of these initial clinical and imaging findings, the working diagnosis was that of an infective or inflammatory process resulting in leptomeningeal and perineural disease, with the differential diagnosis including secondary metastatic deposits or an underlying primary central nervous malignancy. However, an MRI brain study, as well as computed tomography of the thorax, abdomen, and pelvis, was unremarkable, with no other masses or metastatic disease elsewhere. Lumbar puncture was performed, and cerebrospinal fluid analysis did not yield any positive microbiological culture, viral DNA PCR results, or any malignant cells.

**Figure 2:**
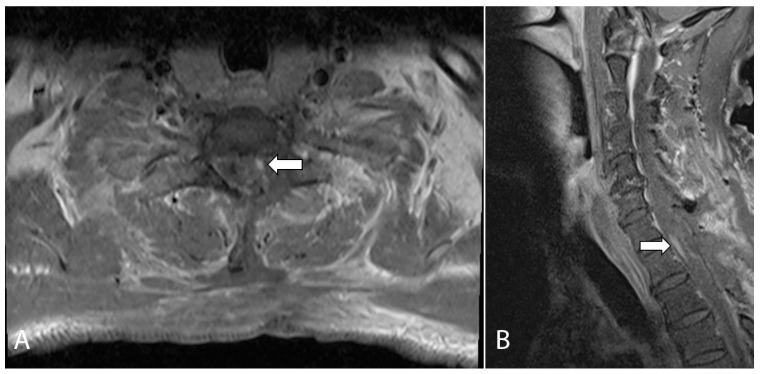
Axial (**A**) and sagittal (**B**) T1-weighted, fat-suppressed, contrast-enhanced MRI of the cervical spine performed on post-operative day one. The images show a small nodular enhancing focus which raised the suspicion of a small focus of residual tumor (arrows).

The patient subsequently underwent C7/T1 hemilaminectomy and excision of the T1 spinal cord lesion and left T1 nerve root. It was noted intraoperatively that there was a hard grayish tumor that was infiltrating along the left T1 nerve root and dorsal column of the spinal cord with both intra- and extramedullary components. Intraoperative frozen sectioning confirmed the presence of a spinal cord and nerve root neoplasm containing spindle and epithelioid cells. MRI performed on the first post-operative day demonstrated a small nodular enhancing focus, which raised the suspicion of residual tumor (Figure 2).

**Figure 3:**
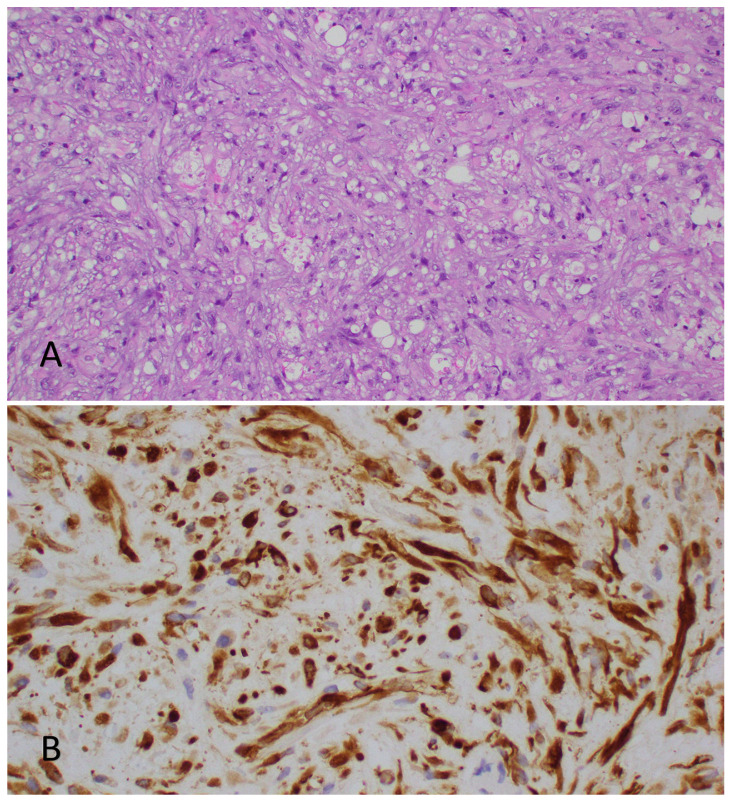
(**A**) Hematoxylin–eosin stain demonstrating spindle cells arranged in fascicles with a vague storiform pattern noted focally. The spindle cells have an irregular nuclear membrane, vesicular chromatin, and occasional prominent nucleoli. Scattered aggregates of cells containing intracytoplasmic vacuoles are present. (**B**) The tumor is also focally positive for cytokeratin AE1/AE3.

On histological analysis, the tumor demonstrated proliferation of both spindle and epithelioid cells with areas of necrosis. The spindle cells were noted to have irregular nuclear membranes, vesicular chromatin, and occasional prominent nucleoli. On immunohistochemical staining, the tumor cells were strongly positive for CAM 5.2 and vimentin, and focally positive for AE1/AE3 and EMA, but negative for CK20, CK7, TTF-1, p63, BerEP4, pCEA, ERG, CD31, and S100 (Figure 3). The proliferative index Ki-67 ranged from 20% to 30%. The tumor was noted to be of unusual morphology and the initial presumptive diagnosis was that of a malignant spindle cell neoplasm, possibly a malignant meningioma with sarcomatoid features. The histology was sent to the MD Anderson Cancer Center for a second opinion, where the morphological features and immunophenotypic profile of the tumor were noted to be consistent with ES.

**Figure 4:**
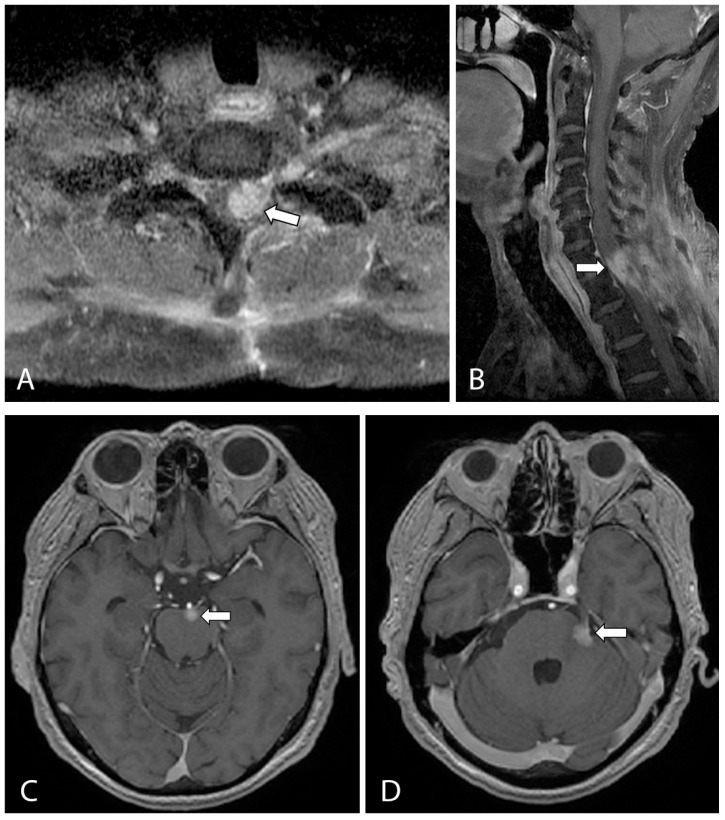
T1-weighted, fat-suppressed, contrast-enhanced axial (**A**) and sagittal (**B**) images from a follow-up MRI cervical spine showing a large enhancing lesion (arrows in (**A**,**B**)) consistent with local recurrence at the levels of C7 and T1, involving the spinal cord and left neural foramen. (**C**,**D**) T1-weighted, contrast-enhanced axial MRI of the brain showed new enhancing lesions at the anterior pons (arrow in (**C**)) and at the left anterolateral surface of the pons (arrow in (**D**)) involving the left trigeminal nerve, which accounted for the new left-sided facial pain.

Post-operative recovery was unremarkable, and adjuvant radiation therapy was started 1 month after surgery. The patient received a total of 33 sessions of radiotherapy over the following 2 months. However, he developed worsening left-sided weakness and new left-sided facial pain within these 2 months. A follow-up MRI spine at 10 weeks post resection demonstrated an enlarging enhancing tumor in the left spinal cord at the levels of C7 and T1 and along the left T1/2 neural foramina. In addition, there were new enhancing lesions along the brainstem and pons, most prominent at the origin of the left trigeminal nerve trunk (Figure 4). Gamma knife surgery was performed for this lesion to reduce the left-sided facial pain.

Clinically, the patient continued to show deteriorating neurological function with progressively worsening bilateral lower-limb weakness and numbness over subsequent months. He also began to develop urinary incontinence and required long-term urinary bladder catheterization. The patient was not keen for further adjuvant chemotherapy or radiotherapy and also declined palliative chemotherapy. He was subsequently referred to palliative medicine and demised within five months from presentation.

## 3. Discussion

ES of the spine is extremely rare. In the reviewed literature, ES typically presented as a large mass in the paravertebral space with or without intraspinal extension [6,7,26,27,28,29,34,35,36,37]. However, in our case, the lesion was predominantly intradural and intramedullary with perineural extension along the T1 nerve root without a large paravertebral component. There have been a few other cases of ES reported within the central nervous system; however, these cases were intracranial ESs: a case of ES of the dura [38] and another case of intracranial ES at the parasagittal location in a patient with known neurofibromatosis type 2 [39]. Our case was noted to have similar histopathological characteristics to the case of ES of the dura. Both cases demonstrated spindle and epithelioid cells with high mitotic activity, exhibited positive immunoreactivity to CAM 5.2, vimentin, and EMA, and were negative for S100. Similarly, neither case demonstrated any meningioma component within the tumor. Other histological differentials included an epithelioid malignant peripheral nerve sheath tumor or synovial sarcoma, although both these entities would typically be positive for S100 [14,38]. A secondary metastatic deposit was also deemed unlikely as the CT thorax, abdomen, and pelvis were negative for malignancy. Ideally, additional immunohistochemical studies including CD34 and INI1 would have been helpful; however, the microscopic and immunohistological features in this case were consistent with those of an ES. Primary central nervous system sarcomas are very rare. These include fibrosarcoma, synovial sarcoma, extraosseous mesenchymal chondrosarcoma, perivascular sarcoma, reticulum cell sarcoma, and myeloid sarcoma [40,41]. To the best of our knowledge, ES arising from the spinal cord has not yet been reported. As previously described, the most common locations for ES are along the limbs (upper or lower extremity) and less commonly in the trunk (in the proximal subtype).

Imaging findings of the tumor in this case showed a predominantly intramedullary lesion with an infiltrative appearance and perineural spread. It was hyperintense on T2-weighted images and hypointense on T1-weighted images, with avid contrast enhancement. In the reviewed literature, MR imaging features of ES showed soft-tissue masses with poorly defined margins and demonstrated growth in a creeping infiltrative manner [42]. Specifically in the spine, ES was seen to be either hypo- or isointense on T1-weighted images with variable homogeneous or heterogeneous signal on T2-weighted images, depending on the degree of necrosis, hemorrhage, or fibrous contents [27]. In our case, the T1-weighted signal and its infiltrative appearance were consistent with known MR imaging findings of ES in the literature. The infiltrative appearance of the tumor in our case again illustrates the propensity for local recurrence. The homogeneous appearance on T2-weighted images could be due to the relatively small size of the tumor without gross central necrosis or hemorrhage. The pattern of metastatic spread is also unusual in our case. The majority of reported spinal ESs demonstrated pulmonary metastatic spread [27], which was not identified in our case. Instead, there was dissemination of disease in the central nervous system. This pattern of metastatic spread could be due to the location of our lesion being predominantly confined within the neuraxis, and not involving the paravertebral soft tissues.

Accurate diagnosis of ES is important as delayed diagnosis is associated with poorer treatment outcomes and prognosis [22,23]. ES can be misdiagnosed initially due to its rarity in clinical practice, and its often-benign initial presentation as a painless slow-growing indolent mass [2,33]. This indolent behavior was also present in our case; the initial diagnostic considerations favored a benign infective or inflammatory process, and the histological diagnosis of a sarcoma was unexpected. Even with tumor histology, the histological diagnosis of ES is challenging due to its rarity, and, in our case, a second opinion report from a high-volume center was required to clinch the diagnosis.

Treatment of spinal ES is more challenging than in other parts of the body due to the presence of surrounding neural structures. In our case, an intra-operative frozen section was obtained despite the indolent tumor behavior to reduce the risk of inadequate resection. However, despite an extensive resection including the left T1 nerve root, there was evidence of inadequate resection on the immediate post-operative MRI with a residual nodular enhancing focus. This highlights the challenge of achieving complete ES resection along the spine.

The tumor in this case exhibited an aggressive behavior, with enlarging local recurrence, metastatic spread, and dissemination within the rest of the central nervous system even while undergoing radiotherapy. Clinically, the patient’s neurological status deteriorated with progressively increasing weakness and numbness in the bilateral lower limbs, due to spinal cord compression from local recurrence. The patient’s clinical course in this case mirrors that in the literature, with a poor overall prognosis, high local recurrence rate, and propensity for metastatic spread.

There was no adjuvant chemotherapy given in our patient. The role of adjuvant chemotherapy in ES remains uncertain in the literature. Existing retrospective studies have suggested that chemotherapy does not have any survival benefit. A long-term multi-institutional retrospective analysis of 55 patients by Kim et al. showed that adjuvant chemotherapy did not influence the time from surgery to recurrence or death [13]. Another retrospective study involving 74 patients with locally advanced or metastatic ES showed that existing conventional systemic chemotherapy (most commonly anthracycline or gemcitabine-based regimens) had an only modest real-world overall response rate of 15% [43]. However, in the systematic review of spinal ES by Hu W. et al., postoperative chemotherapy conferred an increased survival benefit, although the majority of cases were of the proximal subtype. In the study by Kim et al., it was also noted that the proximal subtype of ES benefits more from adjuvant chemotherapy [13]. This suggests that adjuvant chemotherapy could be considered in spinal ES, especially for those of the proximal subtype.

Kim et al. also showed that palliative chemotherapy conferred significantly longer overall survival compared to patients who did not receive chemotherapy, although this was again only observed in patients with the proximal subtype of ES. In our case, given the fast rate of disease progression despite radiotherapy, the decision was made to forego palliative chemotherapy, and the patient opted for hospice care. Overall, the benefit of adjuvant chemotherapy in ES of the spine remains unclear.

## 4. Conclusions

Epithelioid sarcoma (ES) is rare and remains a challenging diagnosis, with different subtypes resulting in varying clinical presentations. Spinal ES is extremely rare with its own unique challenges in diagnosis and treatment. The histological and immunophenotypic features of the tumor described in this case are consistent with ES that occurs at other sites. To the best of our knowledge, spinal ES arising from the spinal cord has not yet been reported. This case illustrates the challenges of early diagnosis of ES, the surgical challenges in achieving adequate resection of spinal ES, and the aggressive behavior of the disease despite adjuvant radiotherapy. The case presented herein expands the topography of ES.

## Data Availability

Not applicable.
